# On the Role of Loopholes in Polite Communication: Linking Subjectivity and Pragmatic Inference

**DOI:** 10.1162/opmi_a_00133

**Published:** 2024-04-10

**Authors:** Nicole Gotzner, Gregory Scontras

**Affiliations:** Cognitive Science Institute, Osnabrück University, Osnabrück, Germany; Department of Language Science, University of California, Irvine, Irvine, CA, USA

**Keywords:** negative strengthening, subjectivity, loopholes, face saving, pragmatics

## Abstract

Existing proposals on the attenuating uses of indirect, negated expressions (e.g., *not happy* to mean *sad*) agree that speakers exploit indirectness for pragmatic purposes but differ on the underlying sources they attribute to these uses. Here, we synthesize existing proposals via adjective subjectivity, which operationalizes the notion of loopholes for plausible deniability. We present experimental evidence that the degree of subjectivity of an adjective predicts the degree to which participants strengthen the negated adjective’s meaning, but only if the adjective under consideration has an evaluatively-positive meaning. This finding indicates that speakers may intentionally use negation to leave themselves the option to retract the implicated face-threatening meaning if openly challenged.

## INTRODUCTION

The attenuating uses of negated expressions like *not happy* have puzzled grammarians and logicians since Greek antiquity (Horn, 1991): why go to the trouble of producing a cumbersome, indirect expression with negation when a more direct expression would suffice to communicate the intended meaning (e.g., *sad*)? Existing proposals agree that speakers exploit negation for pragmatic purposes, but they differ on the underlying sources they attribute to these uses. Speakers may wish to leave themselves a loophole (Seright, 1966), communicate vaguely for plausible deniability (Keenan, 1976; Krifka, 2002), mitigate a face-threatening act (Brown & Levinson, 1987; Horn, 1989, 1991), or simply avoid straightforwardly-negative expressions (e.g., Mazzarella & Gotzner, 2021; Terkourafi et al., 2020; building on the Pollyanna Principle from Boucher & Osgood, 1969). All of these proposals agree that there is an added reward of using these seemingly inefficient utterances, yet the controversy concerns what exactly those benefits are and whether they operate primarily on the speaker’s or the hearer’s side.

Here, we argue that the use of negated expressions is beneficial for both the speaker and the hearer. Specifically, we combine existing proposals via the phenomena of faultless disagreement and polarity: If the simple (i.e., non-negated) utterance the speaker could have used is evaluatively negative (e.g., *Mary is sad*), speakers may opt for a statement involving the negated positive adjective (*Mary is not happy*) to be able to retract the implicated face-threatening, negative meaning if openly challenged. Oftentimes, the use of negation in sentences makes them “so vague that…they defy interpretation” (Givón, 1975, as cited in Israel, 2004). We hypothesize that speakers exploit this indeterminacy in meaning—leaving themselves a loophole for plausible deniability of a straightforwardly-negative, face-threatening meaning—particularly when the alternative simple utterance they could have used is evaluatively negative (e.g., *sad*). The overarching goal of our work is to integrate speaker’s considerations of efficiency, pragmatics, and social rewards in polite language use.

In what follows, we first discuss in more detail the interpretation of negated adjectives, highlighting those cases where the meaning gets strengthened (e.g., *not happy* ⇝ *sad*). We then operationalize the notion of meaning indeterminacy and loopholes via the empirical phenomenon of faultless disagreement, which indexes subjectivity. With these ideas in hand, we present the results of our study investigating the relationship between loopholes and meaning strengthening for negated adjectives. The main prediction of our proposal is that speakers should only have an incentive to communicate via loopholes if the intended meaning is face threatening. Thus, we hypothesize that the degree of an adjective’s subjectivity predicts the interpretation of negated evaluatively-positive adjectives (*not happy*) but not of evaluatively-negative ones (*not sad*).

## THEORETICAL BACKGROUND

### Accounts of Negative Strengthening

Negation has an attenuating effect on meaning, which does not follow from classical propositional logic but rather from pragmatic considerations underlying the use of negation (Horn, 1989; Israel, 2004). A key puzzle in the literature pertains to the asymmetric interpretation of negated adjectives, as shown in (1), where we see that negated evaluatively-positive adjectives like *happy* are more likely to have their meanings strengthened, (1a), than negated evaluatively-negative adjectives like *sad*, (1b).

(1)  a. Mary is not happy ⇝ Mary is sad
    b. Mary is not sad 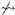 Mary is happy

The most prominent account of this asymmetry is based on politeness theory. On this view, the speaker uses a negated adjective to mitigate the face threat posed to the hearer by a simple adjective that conveys a negative evaluation. Consider a speaker saying that *Mary is not happy* when Mary is in fact sad. The use of *not happy* may be considered more polite, as the direct expression of the simple antonym *sad* is evaluatively negative (Brown & Levinson, 1987; Horn, 1989, 1991). The hearer, in turn, can recognize this reasoning and interpret *not happy* as *sad*. In this case, the meaning of the negated expression gets pragmatically strengthened, thus the term “negative strengthening”.

Evidence for the politeness-based account comes from the role of polarity (see Colston, 1999; Fraenkel & Schul, 2008; Ruytenbeek et al., 2017) and sociological variables (Gotzner & Mazzarella, 2021) in the interpretation of negated adjectives. Negative strengthening is typically available for evaluatively-positive adjectives but not for negative ones, as shown in (1). The absence of negative strengthening for evaluatively-negative adjectives is due to the fact that one can simply say that *Mary is happy* without threatening the addressee’s face. Typically, there is no reason to avoid the direct expression of a positive evaluation—unless the speaker is concerned that the positive evaluation would communicate additional unintended meaning (e.g., affection; Horn, 1991; Gotzner & Mazzarella, 2021).^1^

The politeness-based account motivates the use of negated expressions to save the hearer’s face. An alternative account of the polarity asymmetry is centered around considerations wholly internal to the speaker. This account is based on the Pollyanna Principle, which states that there is a “universal human tendency to use positive words more frequently” (Boucher & Osgood, 1969; Dodds et al., 2015). As proposed by Terkourafi et al. (2020) and Mazzarella and Gotzner (2021), this principle could be operative in the use of negated expressions, thereby leading the speaker to avoid straightforwardly-negative evaluations. This avoidance may be motivated by the desire to focus on the bright side of life because it makes the speaker feel better independently of concerns about the hearer’s feelings. The use of positive words may put the speaker in a good emotional state or may help save their own face (see Brown & Levinson, 1987; Yoon et al., 2020, for more on self-presentational concerns of the speaker). In either case, the Pollyanna principle could be operative without any recursive reasoning about the effect of an utterance on the hearer. In line with this view, Mazzarella and Gotzner (2021) demonstrated the polarity asymmetry as in (1) even in contexts that do not pose a face threat to the addressee.

A final proposal in the literature revolves around the notion of loopholes and plausible deniability (Keenan, 1976; Krifka, 2002; Pinker et al., 2008; Seright, 1966) and this account, too, focuses on the speaker’s side. When using a negated expression, the speaker is able to retract the implicated meaning. *Not happy* opens a number of interpretative options and therefore does not commit the speaker to conveying *sad*. Thus, the speaker is able to hedge and retract the implicated meaning if openly challenged, as shown in (2).

(2)  Greg: Mary is not happy.
    Nicole: You said that Mary sad.
    Greg: No, I never said that!

To summarize, there are several potential reasons to avoid a direct expression of *sad*: (i) it would pose a face-threat to the hearer and (ii) the speaker prefers to express positive meanings. On the other hand, there is an incentive for the speaker to use a negated expression because it leaves them the option to retract the implicated meaning. The three accounts in the literature focus on different pragmatic reasoning strategies involving the speaker’s vs. hearer’s concerns. Table 1 summarizes the different principles proposed by the three different accounts while also specifying the side of the communication dyad—the speaker or the hearer—on which they primarily operate.

**Table 1.** Summary of different accounts of negative strengthening.

	**Politeness**	**Pollyanna**	**Loophole**
Principle	avoid face threat	stay positive	remain vague
Side	hearer	speaker	speaker

While there exists quantitative empirical evidence in support of the politeness and Polyanna proposals, there is less evidence for reasoning about loopholes for the purpose of negative strengthening. Our study aims to find such evidence for the role of loopholes while also refining the loophole proposal to take into account considerations of polarity. Rather than incentivizing loopholes across the board for both evaluatively-positive and evaluatively-negative adjectives, we suspect that speakers strategically exploit loopholes only if the conveyed meaning may be face-threatening. In other words, loopholes are useful to the extent that they allow the speaker to avoid face threat, or at least retract a face-threatening meaning if challenged. Thus, we expect to find that speaker’s uses of loopholes are sensitive to evaluative polarity.

In order to make progress on understanding the role of loopholes, in the following subsection we make explicit our assumptions regarding what loopholes are and how they operationalize behaviorally.

### Subjectivity

We have talked about loopholes as opening up a number of interpretive options, which then allow the speaker to retract a face-threatening, negative meaning. To make more precise this notion of interpretive optionality, here we conceive of loopholes in terms of the notion of subjectivity. While many pieces of language express a more precise, more objective meaning (consider *mother*, *alive*, or *in Germany*), others express meanings that are less precise, or more subjective. The source of the subjectivity may be vagueness (e.g., *happy* by which standard?), evaluativity (e.g., *beautiful* according to whom?), or context dependence (e.g., *large* compared to what?). Regardless of the underlying cause, the result is that certain pieces of language are perceived to be more subjective than others, such that speakers and listeners cannot always be sure that they are understanding language in the same way. The potential for misalignments in understanding that arises as a result of inter-speaker subjectivity creates the loophole by which speakers may deny an unpalatable meaning.

A prominent way of evaluating the subjectivity of language is the faultless disagreement task (Barker, 2013; Kennedy, 2013; Kölbel, 2004; Solt, 2016), which captures potential uncertainty about assessment criteria and outcomes. In the task, people encounter a disagreement between two speakers, as in (3):

(3)  Greg: Mary is not sad.
    Nicole: Mary is sad.

The task is to decide whether the two speakers could both be right while uttering their disagreeing statements, or whether one of the speakers must be wrong. To the extent that the two speakers in (3) can both be right while disagreeing with their statements, the adjective *sad* admits that degree of faultless disagreement; for our purposes, faultless disgreement operationalizes adjective subjectivity. While seemingly simple, this task offers a powerful tool for understanding nuances of adjective meaning (e.g., Solt, 2016), given that different adjectives admit different degrees of faultless disagreement.

**Figure 1.**
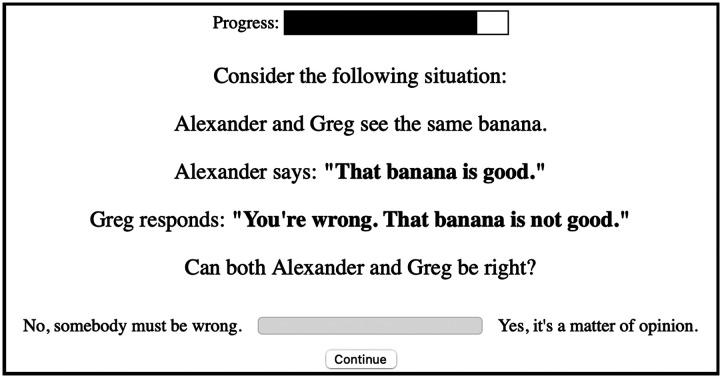
Example trial from Expt. 1: *Faultless disagreement* from Scontras et al. (2017).

**Figure 2.**
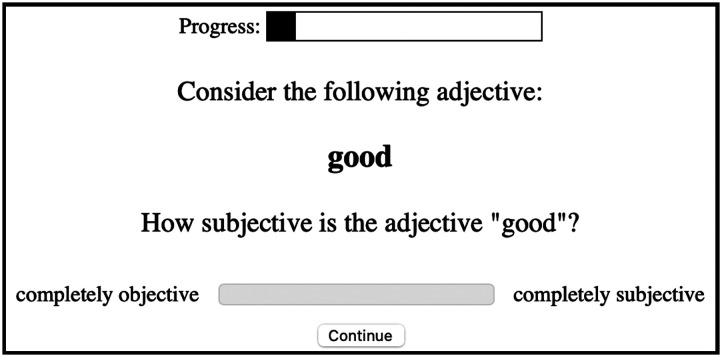
Example trial from Expt. 1: *Subjectivity* from Scontras et al. (2017).

Importantly, naive experimental participants have reliable judgments of faultless disagreement, and these judgments align with independent measures of subjectivity. In their investigation of the preferences determining the relative placement of adjectives in English (e.g., *big blue box* vs. *blue big box*), Scontras et al. (2017) conducted a faultless disagreement task for 26 English adjectives (Figure 1), as well as a separate task asking participants to directly rate the “subjectivity” of the same 26 adjectives (Figure 2). The two measures were highly correlated (*r*^2^ = 0.89), suggesting that naive experimental participants interpret “subjectivity” as intended, namely in terms of the potential for faultless disagreement. Color, material, and shape adjectives were found to have low faultless disagreement/subjectivity scores, while size and quality adjectives had much higher scores. For our purposes, subjectivity as operationalized via faultless disagreement also operationalizes the notion of loopholes in polite communication.

## CURRENT STUDY: THE ROLE OF SUBJECTIVITY IN NEGATIVE STRENGTHENING

The existing accounts of negative strengthening provide at least two disincentives for using a more direct negative expression like *sad*—as opposed to the less direct negated expression *not happy*. To the extent that speakers are concerned about their listeners, they should avoid the evaluatively-negative face-threatening meaning that is associated with *sad*. To the extent that speakers simply avoid straightforwardly-negative expressions à la the Pollyanna Principle, they should also avoid *sad*. On the other hand, there is an incentive for the speaker to use a negated expression because it is less specific and it leaves the speaker a loophole. Even though the process of negative strengthening may lead a negated expression like *not happy* to mean sad, as shown in (2), speakers may wish to retract when the implicated meaning is evaluatively negative.

We thus synthesize the different accounts of negative strengthening by combining loopholes with evaluative polarity and politeness considerations. Speakers should exploit loopholes when the implicated meaning is negative. For positive evaluations, there is no straightforward reason to use a hedged negated expression (e.g., *not sad*) as opposed to a direct expression (e.g., *happy*). Thus, speakers should be less likely to exploit loopholes when the implicated meaning is evaluatively positive than when it is evaluatively negative.

We operationalize loopholes as the potential for faultless disagreement of an adjective, which indexes adjective subjectivity. The higher the degree of subjectivity, the greater the potential for a loophole. On the comprehender’s side, when they encounter a negated adjective, to the extent that the adjective is subjective (thereby opening up a loophole for the speaker), the comprehender will arrive at a higher degree of negative strengthening. Crucially, this reasoning applies only to negated positive adjectives (*not happy*), where the speaker faces disincentives to avoid the straightforward expression of the evaluatively-negative meaning (*sad*); subjectivity should not impact the interpretation of negated evaluatively-negative adjectives in the same way.

To investigate the relationship between loopholes and negative strengthening, we need two ingredients: (i) we need measures of negative strengthening for a range of both evaluatively-positive and evaluatively-negative adjectives, which will come from the negative strengthening scores collected by Mazzarella and Gotzner (2021); and (ii) we need subjectivity scores for those adjectives, which we will collect. Our pre-registered hypothesis is that the degree of negative strengthening for evaluatively-positive adjectives is predicted by subjectivity scores, while the interpretation of negated negatives may be subject to other pragmatic considerations. We therefore predict an interaction betwen subjectivity and polarity such that negative strengthening is more likely to occur as adjective subjectivity increases—but only for positive adjectives.

### Methods

#### Items.

Table 2 presents the adjectives we used in our study, which come from Mazzarella and Gotzner (2021) (building on French materials from Ruytenbeek et al., 2017). Adjectives were paired: an evaluatively-positive adjective (e.g., *happy*, *strong*, *lucky*) and its corresponding negative antonym (e.g., *sad, weak, unlucky*). Ruytenbeek et al. (2017) normed the pairs for a range of linguistic tests such as whether the adjective under consideration is evaluatively positive or negative. Mazzarella and Gotzner verified that English translation equivalents had the same polarity and selected those pairs with consistent criteria. In total, the materials consist of 40 adjectives (see Appendix A of Mazzarella & Gotzner, 2021 for more information on the items).

**Table 2.** Twenty antonym pairs from Gotzner and Mazzarella (2021) used in the current study.

**positive**	**negative**	**positive**	**negative**
certain	uncertain	useful	useless
lucky	unlucky	good	bad
accurate	inaccurate	strong	weak
happy	unhappy	kind	mean
interesting	uninteresting	tall	short
fair	unfair	happy	sad
polite	impolite	long	short
possible	impossible	polite	rude
satisfactory	unsatisfactory	rich	poor
friendly	unfriendly	satisfactory	frustrating

#### Negative Strengthening Ratings.

**Figure 3.**
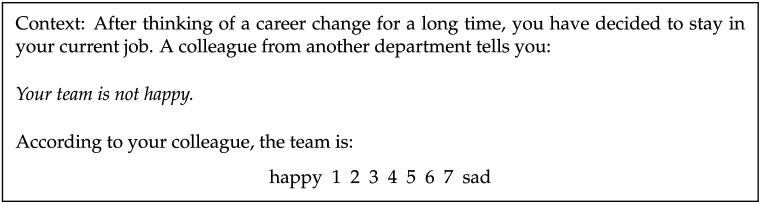
Example trial from Mazzarella and Gotzner (2021) measuring negative strengthening.

Our assessments of negative strengthening come from the combined results of Mazzarella and Gotzner (n = 140), who embedded the same 20 antonym pairs in discourses of the type in Figure 3.^2^ Following a context, a statement with a negated adjective was presented. Participants were asked to judge whether the speaker intended to convey the meaning communicated by the non-negated adjective or the meaning associated with its antonym (e.g., whether, according to the speaker, *not happy* conveys happy vs. sad), using a seven-point scale. The scale was anchored at the adjective in the critical negated statement, thus measuring the degree of negative strengthening as a function of the likelihood that the antonym is taken to be conveyed by the speaker’s utterance. Higher ratings indicate a greater degree of negative strengthening. The study used a Latin-square design, so a given participant judged one adjective of an antonym pair (e.g., *rich* or *poor* but not both).

The data from Mazzarella and Gotzner (2021) are publicly available on the following repository: osf.io/q3g2n/. We used the same data and main model for our analysis, while adding adjective subjectivity as an additional covariate (see Table 3).

**Table 3.** Cumulative link mixed effects model: clmm(neg-strengthening ∼ polarity * adj-subjectivity + (1 + polarity∣item) + (1 + polarity∣participant).

Random effects:				
	Variance	Corr	Corr	
participant	5.4446	2.3334		
polarity	1.4439	1.2016	−0.448	
item	0.162	0.4025		
polarity	0.2049	0.4526	−0.838	
Coefficients:				
	Estimate	SE	*z*-value	*P*-value
polarity	0.46788	0.08282	5.649	0.0001
subjectivity	0.53069	0.55679	0.953	0.3405
polarity:subjectivity	1.4541	0.56221	2.586	0.0097

#### Subjectivity Ratings.

To measure adjective subjectivity, we used the methodology from Expt. 1: *Subjectivity* from Scontras et al. (2017). 60 English-speaking participants encountered a series of 20 antonym pairs and rated their subjectivity on a sliding scale with endpoints labeled “completely objective” (coded as 0) and “completely subjective” (coded as 1). We measured subjectivity for four elements: the positive adjective (e.g., *happy*), the negated positive (e.g., *not happy*), the negative antonym (e.g., *sad*), and the negated negative (e.g., *not sad*); participants saw one version of each item at random.^3^ Subjectivity scores were averaged across participants and used in the subsequent analysis. An example trial is presented in Figure 2.

### Results

**Figure 4.**
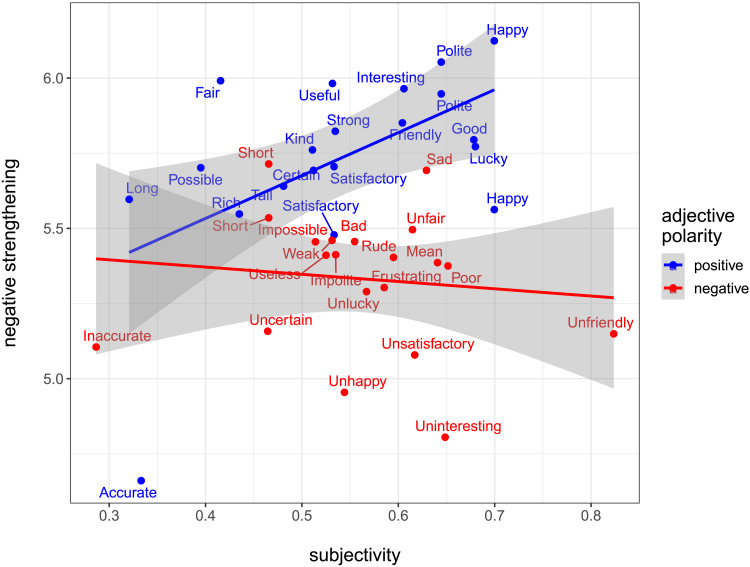
Negative strengthening scores (from Mazzarella & Gotzner, 2021) plotted against the subjectivity scores for the adjectives tested in our study. Note that some adjectives were used twice but paired with a different antonym.

To assess the role of subjectivity in the negative strengthening data, we used our average subjectivity scores by adjective to predict negative strengthening. Our preregistered hypothesis is that subjectivity ratings predict negative strengthening for negated evaluatively-positive adjectives but not evaluatively-negative ones. Hence, there should be an interaction between subjectivity and polarity. Figure 4 displays the effect of subjectivity on negative strengthening, grouped by the polarity of the terms involved. We fit a mixed-effects cumulative link model predicting negative strengthening with the factors polarity (positive vs. negative), centered subjectivity scores, and an interaction of the two factors, together with the maximal random effects structure justified by our design (see Table 3). The polarity effect originally found in the data by Mazzarella and Gotzner persists in the model: positive-polarity adjectives yield more negative strengthening than negative adjectives (*β* = 0.47, *z* = 5.65, *p* < 0.001). We further find an interaction of polarity and subjectivity (*β* = 1.45, *z* = 2.59, *p* < 0.01). As predicted, and as shown in Figure 4, with negated positive-polarity adjectives, negative strengthening increases with increased subjectivity (*r* = 0.54; *r*^2^ = 0.30, 95% CI [0.0035, 0.6166]); with negative-polarity adjectives, there is no correlation (*r* = −0.11; *r*^2^ = 0.01, 95% CI [0.000, 0.127]).^4^ All results and analysis scripts, together with our preregistered hypothesis, can be found on the OSF repository: https://osf.io/nm52h/.

In response to this finding, reviewers expressed skepticism that our direct method of evaluating subjectivity offers a convincing operationalization of loopholes for the purpose of the current investigation. To address this worry, we separately measured subjectivity using the arguably more ecologically valid faultless disagreement task from Scontras et al. (2017), as exemplified in Figure 1. Using faultless disagreement scores as our measure of subjectivity, we replicate the crucial interaction between adjective polarity and subjectivity (*β* = 1.19, *z* = 3.26, *p* < .01): negative strengthening increases with subjectivity scores for positive adjectives. Additional details of the faultless disagreement task and analysis can be found on the OSF repository.

## DISCUSSION

We have investigated the interpretation of negated adjectives by synthesizing existing proposals via an adjective’s subjectivity and its evaluative polarity. Our hypothesis was that speakers exploit loopholes when the implicated meaning is negative to safe-guard the addressee’s face. We thus predicted that participants are more likely to strengthen an adjective under negation as the potential for loopholes—as operationalized by adjective subjectivity—increases. In turn, speakers should not exploit such a strategy to deny an implicated positive meaning and thus the degree of adjective subjectivity should not play a role for negated evaluatively-negative adjectives.

Our predicted interaction between polarity and subjectivity was borne out. This finding is consistent with the idea that speakers may intentionally use negation to leave themselves the option to retract the implicated face-threatening meaning if openly challenged, thereby avoiding the direct expression of something negative.

Our finding suggests that there are politeness considerations that would lead the speaker to not want to directly communicate *sad*; the listener observes the speaker is using a negated term (*not happy*), which signals to the listener that the speaker may be trying to be polite or keep things positive; the listener corrects for the speaker’s behavior and interprets the negated term as worse than it otherwise would be (literally) interpreted. The idea that speakers may want to leave room for deniability is well-attested in the literature. However, a strict reading of the existing proposals (e.g., Seright, 1966) might lead one to expect only a main effect of subjectivity, such that more subjective adjectives lead to more negative strengthening across the board. Our finding of an interaction between subjectivity and polarity suggests that there is more to the story than simply leaving loopholes: loopholes are useful in situations where the message that would be communicated is potentially face-threatening to the listener or otherwise undesirable to the speaker because of its negativity. That is, only when speakers use a negated positive do we find this pressure toward loopholes, as evidenced in the correlation with subjectivity.

It is important to note that our appeal to politeness is compatible with speakers who may not be wholly altruistic, avoiding the hurt feelings of their listeners. Instead, speakers may be avoiding negative words (and their corresponding meanings) for selfish reasons, simply to avoid the taboo associated with those words or because it makes them feel better (see also Mazzarella & Gotzner, 2021; Terkourafi et al., 2020). In other words, speakers may be concerned primarily with their own face, rather than the hearer’s. Speakers have self-representational concerns about their own face, for example they want to appear pro-social (Yoon et al., 2020). Brown and Levinson (1987) argue that off-record politeness strategies allow the speaker to mitigate a face-threatening an act, be perceived as tactful, and open up the opportunity to evade responsibility. We see it as an interesting avenue for future work to tease apart the different underlying motivations of the speaker. Speakers may sometimes remain vague for prosocial reasons and in other contexts for selfish reasons (see also Vallauri, 2019). Regardless of the exact motivation underlying the speaker’s use of negated adjectives, our finding relating negative strengthening with subjectivity for positive adjectives points to the role of faultless disagreement in the use of negated (positive) adjectives. Since faultless disagreement and loopholes concern a potential challenge by a hearer, we do believe that speakers take the hearer’s perspective into account. We thus conclude that an account of the polarity asymmetry has to take both speakers’ concerns about efficiency and loopholes as well as their concerns about the effects of their language on the hearer into account.

Overall, our work adds to the growing literature which indicates that pragmatic strengthening is subject to both the semantic characteristics of the adjectives used (i.e., polarity and perceived subjectivity) as well as to broader social considerations. In the process, we find further support for the idea that, although indirect speech may appear to be inefficient, it plays a key role in managing social relationships (see for example Pinker et al., 2008).

## ACKNOWLEDGMENTS

We thank the audience of XPRAG 2023 in Paris as well as Kristina Kobrock and Noa Attali for valuable feedback on this work.

## FUNDING INFORMATION

Our research was supported by the DFG (International collaboration grant GO 3378/4-1, project number 523308575).

## DATA AVAILABILITY STATEMENT

All data and code are publicly available: https://osf.io/nm52h/.

## Notes

^1^ While most scholars have taken the role of evaluative polarity as an index of social factors, some accounts outline pragmatic principles related to markedness to explain the asymmetric interpretation of negated statements (e.g., Krifka, 2007; Ruytenbeek et al., 2017). Often, negated statements are interpreted as weaker compared to their simple counterparts (see Horn, 1989; Gotzner & Kiziltan, 2022, for a direct experimental comparison).^2^ Mazzarella and Gotzner (2021) present the results of two experiments with contextual manipulation of the face-threatening potential of the adjectives. The study found a main effect of polarity in negative strengthening and no effect of context. Thus, we combine the results of the two experiments for our analysis. The study also replicated an interaction of polarity and morphological complexity found by Ruytenbeek et al. (2017). Details on these analyses as well as all contexts and items are provided in the Appendices of Mazzarella and Gotzner (2021), available at https://www.glossa-journal.org/article/id/5427/.^3^ We included the negated adjectives so that we could calculate a subjectivity difference score by item: subtracting the subjectivity of a negated adjective from that of the corresponding antonym. As we later detected, there is a statistical and conceptual issue with this measure. See Footnote 4 for more on these difference scores.^4^ When we exclude the outlier item *accurate–inaccurate*, we still observe a main effect of polarity and an interaction of polarity with subjectivity. We ran a further analysis with the difference score subtracting the subjectivity of the negated adjective from that of the corresponding antonym; however, this analysis did not yield significant results. Because the subjectivity score for negated adjectives is correlated with the subjectivity of its antonym, most of the difference scores are close to zero; thus, we lose information about the relative subjectivity across items in this difference-score analysis.

## References

[bib1] Barker, C. (2013). Negotiating taste. Inquiry, 56(2–3), 240–257. 10.1080/0020174X.2013.784482

[bib2] Boucher, J., & Osgood, C. E. (1969). The Pollyanna hypothesis. Journal of Verbal Learning and Verbal Behavior, 8(1), 1–8. 10.1016/S0022-5371(69)80002-2

[bib3] Brown, P., & Levinson, S. C. (1987). Politeness: Some universals in language usage. Cambridge University Press. 10.1017/CBO9780511813085

[bib123] Colston, H. (1999). “Not good” is “bad”, but “not bad“ is not “good”: An analysis of three accounts of negation asymmetry. Discourse Processes, 28(3), 237–256. 10.1080/01638539909545083

[bib4] Dodds, P. S., Clark, E. M., Desu, S., Frank, M. R., Reagan, A. J., Williams, J. R., Mitchell, L., Harris, K. D., Kloumann, I. M., Bagrow, J. P., Megerdoomian, K., McMahon, M. T., Tivnan, B. F., & Danforth, C. M. (2015). Human language reveals a universal positivity bias. Proceedings of the National Academy of Sciences, 112(8), 2389–2394. 10.1073/pnas.1411678112, 25675475 PMC4345622

[bib124] Fraenkel, T., & Schul, Y. (2008). The meaning of negated adjectives. Intercultural Pragmatics, 5(4), 517–540. 10.1515/IPRG.2008.025

[bib5] Givón, T. (1975). Negation in language: Pragmatics, function, ontology. Syntax and Semantics, 9, 69–112.

[bib6] Gotzner, N., & Kiziltan, S. (2022). She is brilliant! Distinguishing different readings of relative adjectives. In N. Gotzner & U. Sauerland (Eds.), Measurement, numerals and scales (pp. 117–134). Palgrave Macmillan. 10.1007/978-3-030-73323-0_7

[bib7] Gotzner, N., & Mazzarella, D. (2021). Face management and negative strengthening: The role of power relations, social distance, and gender. Frontiers in Psychology, 12, 602977, 10.3389/fpsyg.2021.602977, 34646182 PMC8502883

[bib8] Horn, L. R. (1989). A natural history of negation. Univeristy of Chicago Press.

[bib9] Horn, L. R. (1991). *Duplex negatio affirmat …*: The economy of double negation. In L. M. Dorbin, L. Nichols, & R. M. Rodriguez (Eds.), CLS 27-II: Papers from the parasession on negation (pp. 80–106). Chicago Linguistic Society.

[bib10] Israel, M. (2004). The pragmatics of polarity. In L. R. Horn & G. Ward (Eds.), The handbook of pragmatics (pp. 701–723). Blackwell. 10.1002/9780470756959.ch31

[bib11] Keenan, E. O. (1976). The universality of conversational postulates. Language in Society, 5(1), 67–80. 10.1017/S0047404500006850

[bib12] Kennedy, C. (2013). Two sources of subjectivity: Qualitative assessment and dimensional uncertainty. Inquiry, 56(2–3), 258–277. 10.1080/0020174X.2013.784483

[bib13] Kölbel, M. (2004). Faultless disagreement. Proceedings of the Aristotelian Society, 104, 53–73. 10.1111/j.0066-7373.2004.00081.x

[bib14] Krifka, M. (2002). Be brief and vague! And how Bidirectional Optimality Theory allows for verbosity and precision. In D. Restle & D. Zaefferer (Eds.), Sounds and systems: Studies in structure and change. A festschrift for Theo Vennemann (pp. 439–458). De Gruyter Mouton. 10.1515/9783110894653.439

[bib50] Krifka, M. (2007). Negated antonyms: Creating and filling the gap. In U. Sauerland & P. Stateva (Eds.), Presupposition and implicature in compositional semantics (pp. 163–177). Palgrave Macmillan. 10.1057/9780230210752_6

[bib15] Mazzarella, D., & Gotzner, N. (2021). The polarity asymmetry of negative strengthening: Dissociating adjectival polarity from facethreatening potential. Glossa: A Journal of General Linguistics, 6(1), 47. 10.5334/gjgl.1342

[bib16] Pinker, S., Nowak, M. A., & Lee, J. J. (2008). The logic of indirect speech. Proceedings of the National Academy of Sciences, 105(3), 833–838. 10.1073/pnas.0707192105, 18199841 PMC2242675

[bib17] Ruytenbeek, N., Verheyen, S., & Spector, B. (2017). Asymmetric inference towards the antonym: Experiments into the polarity and morphology of negated adjectives. Glossa: A Journal of General Linguistics, 2(1), 92. 10.5334/gjgl.151

[bib18] Scontras, G., Degen, J., & Goodman, N. D. (2017). Subjectivity predicts adjective ordering preferences. Open Mind: Discoveries in Cognitive Science, 1(1), 53–66. 10.1162/OPMI_a_00005

[bib19] Seright, O. D. (1966). Double negatives in standard modern English. American Speech, 41, 123–126. 10.2307/453131

[bib20] Solt, S. (2016). Ordering subjectivity and the absolute/relative distinction. In N. Bade, P. Berezovskaya, & A. Schöller (Eds.), Proceedings of Sinn und Bedeutung 20 (pp. 676–693).

[bib21] Terkourafi, M., Weissman, B., & Roy, J. (2020). Different scalar terms are affected by face differently. International Review of Pragmatics, 12(1), 1–43. 10.1163/18773109-01201103

[bib22] Vallauri, E. L. (2019). La lingua disonesta. Il Mulino.

[bib23] Yoon, E. J., Tessler, M. H., Goodman, N. D., & Frank, M. C. (2020). Polite speech emerges from competing social goals. Open Mind: Discoveries in Cognitive Science, 4, 71–87. 10.1162/opmi_a_00035, 33225196 PMC7672308

